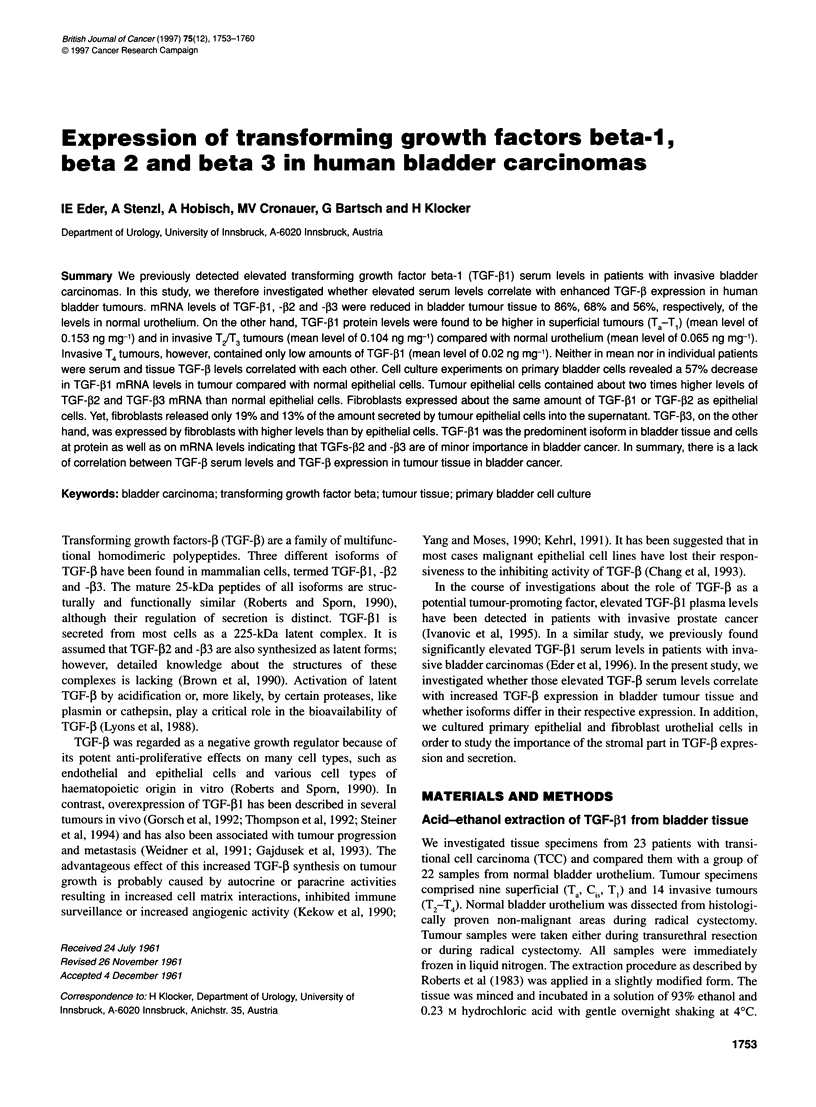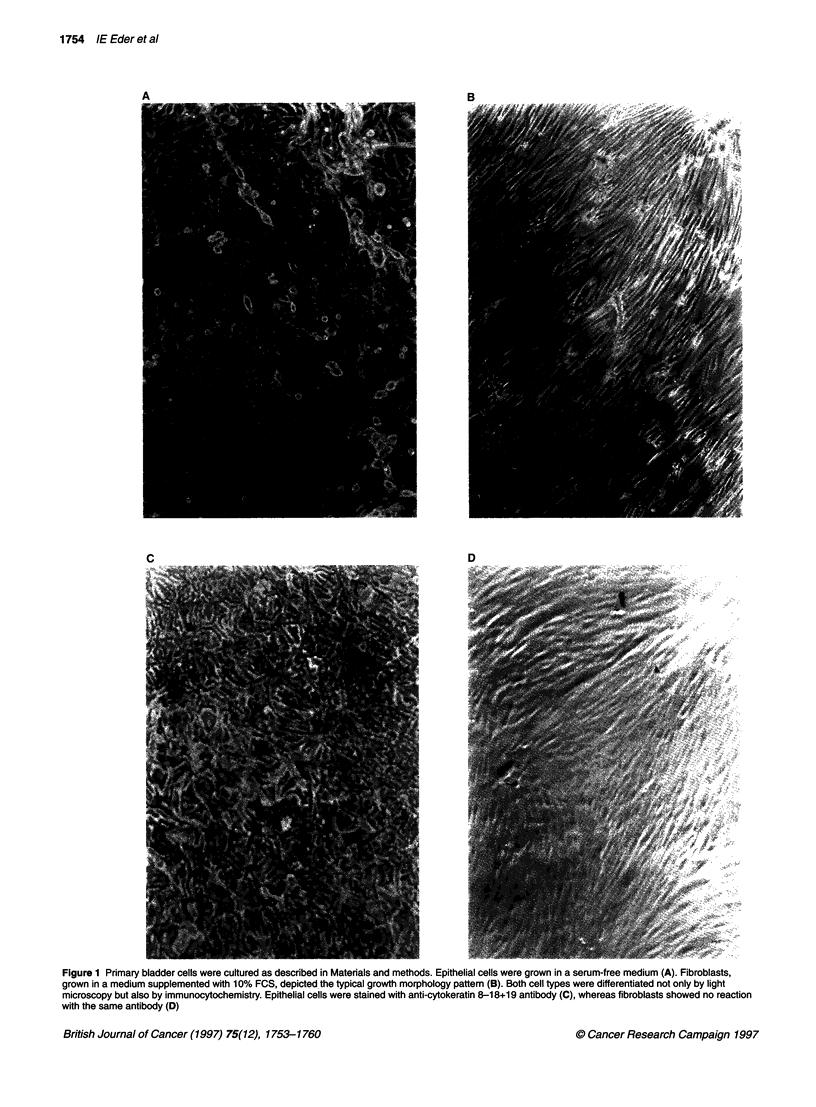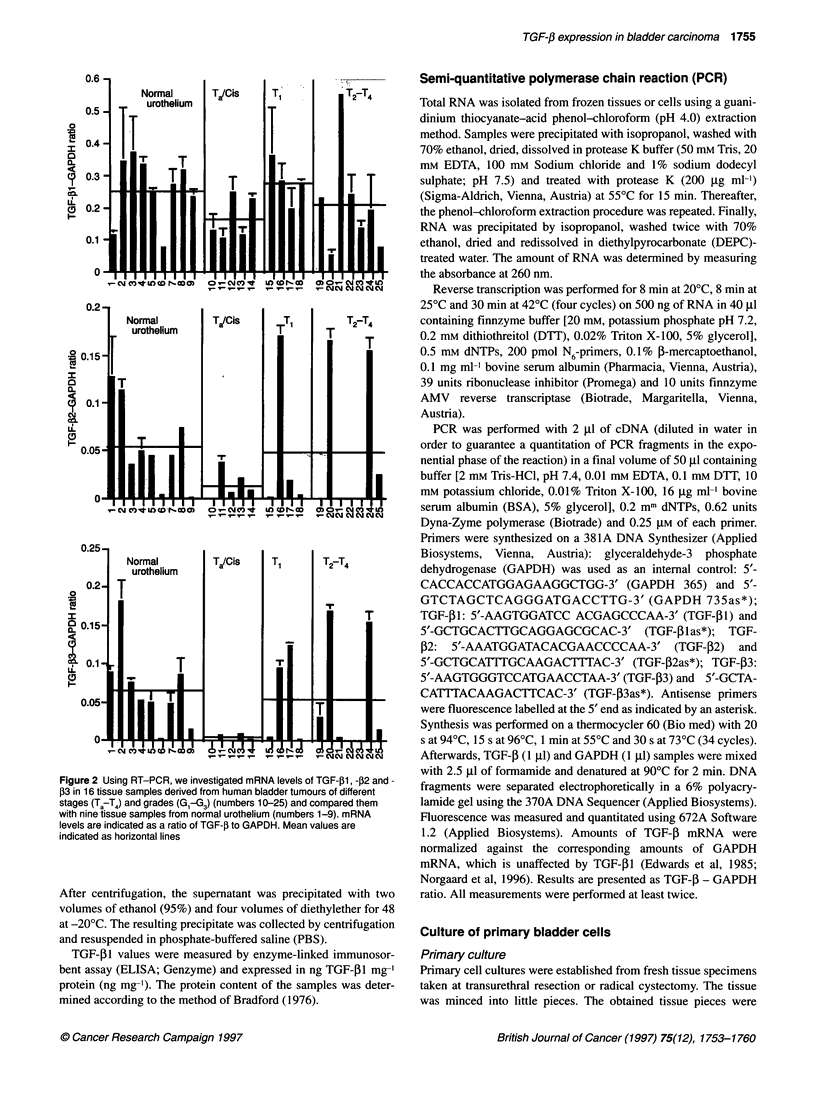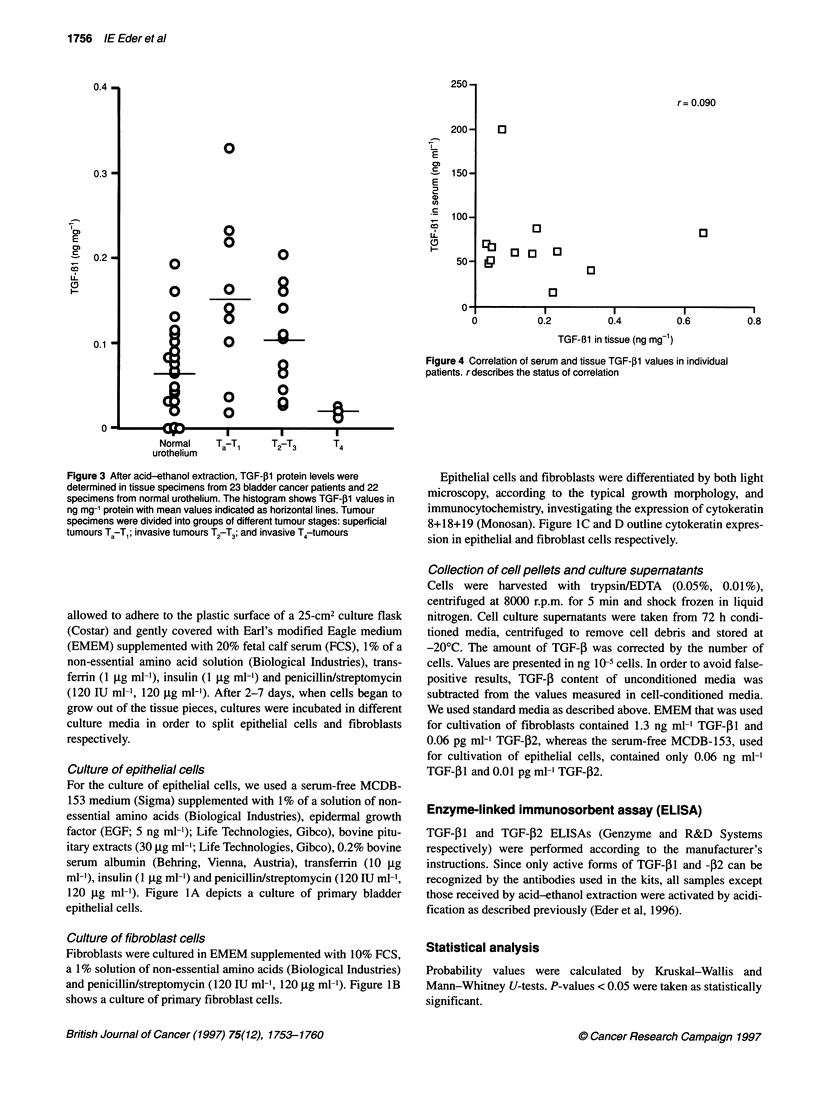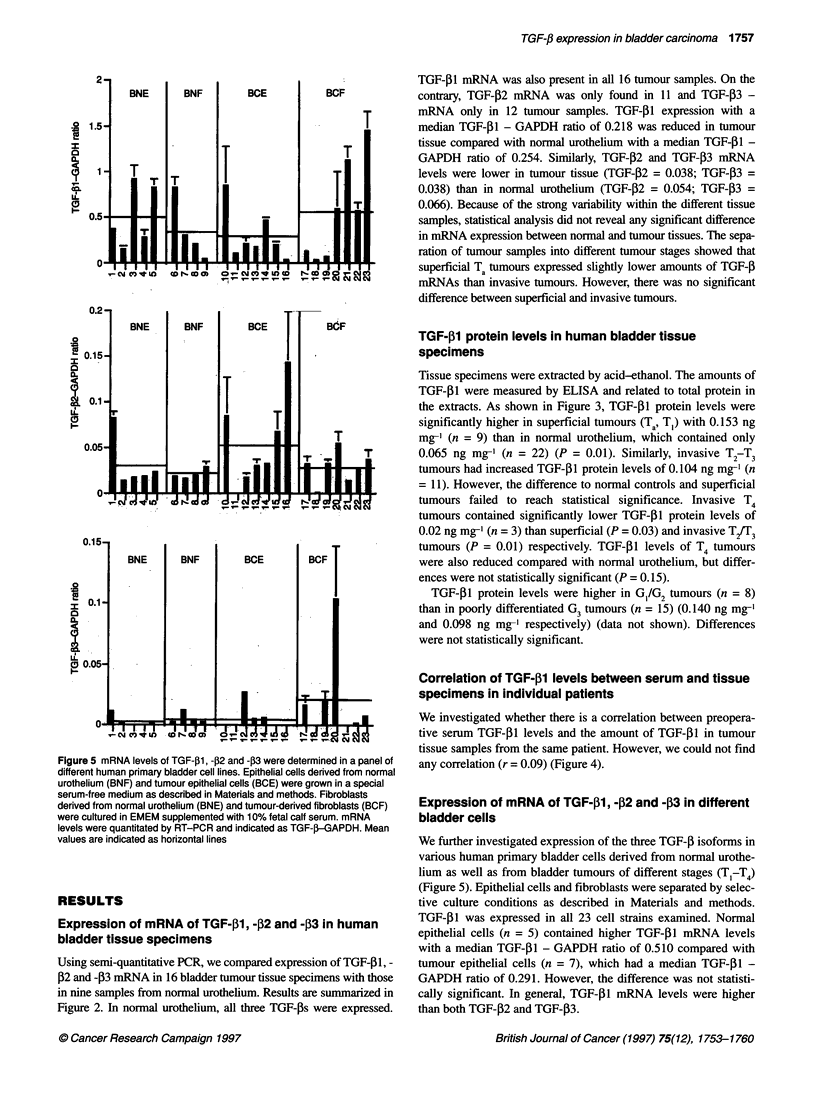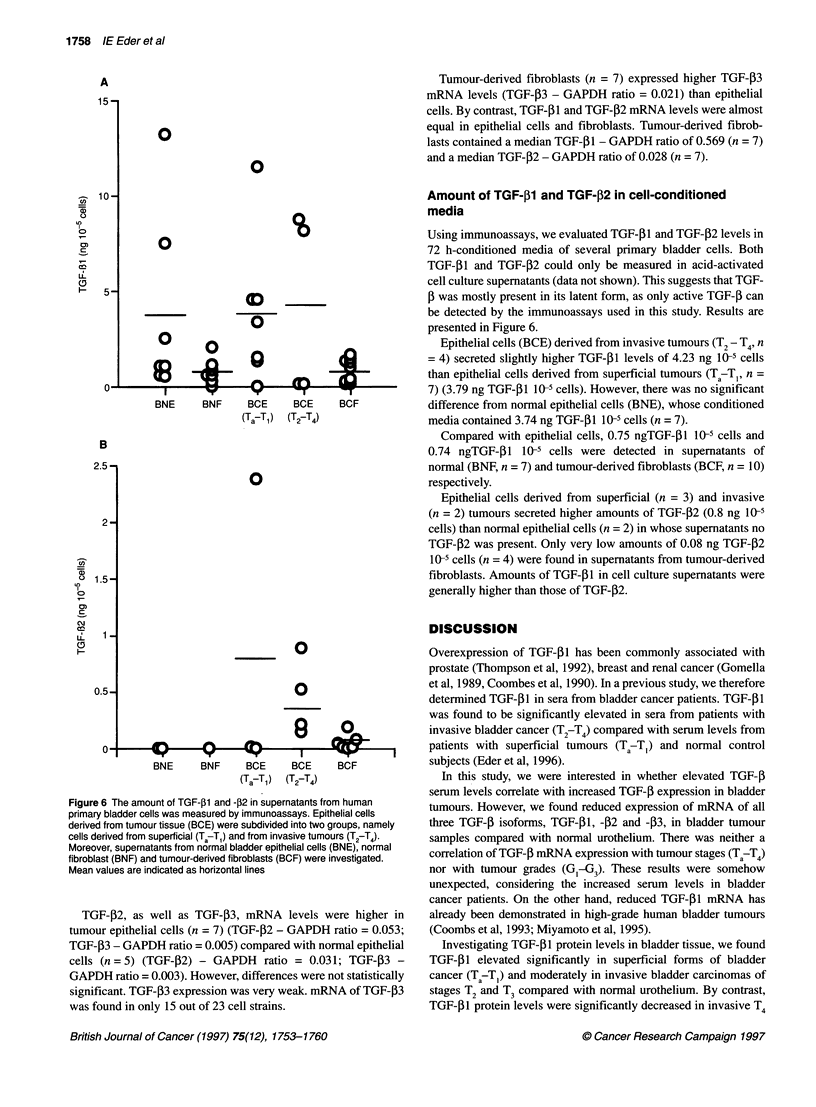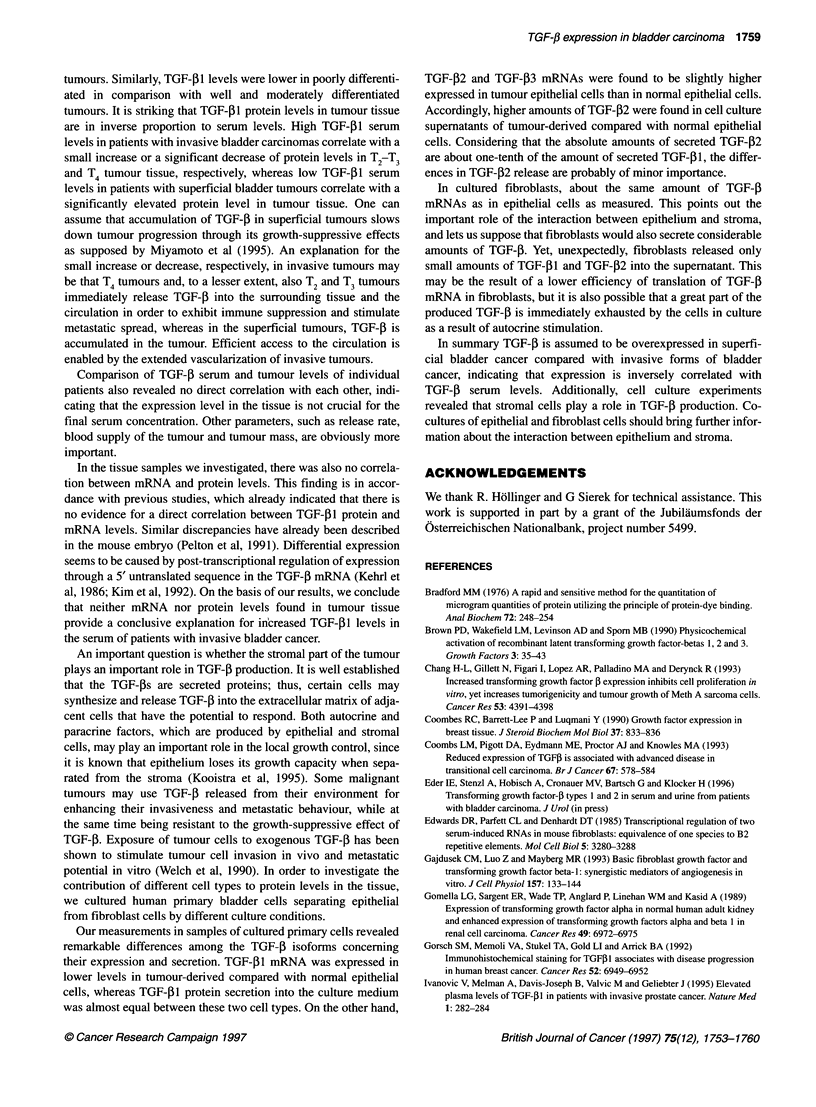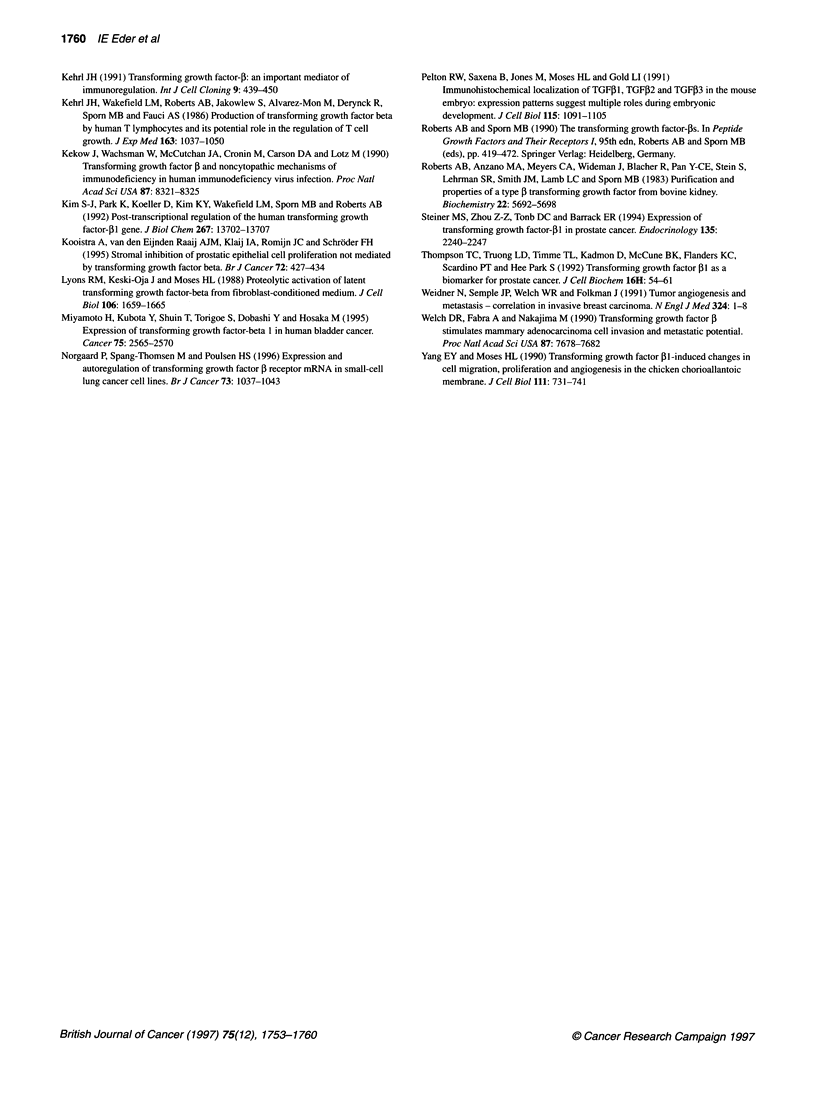# Expression of transforming growth factors beta-1, beta 2 and beta 3 in human bladder carcinomas.

**DOI:** 10.1038/bjc.1997.299

**Published:** 1997

**Authors:** I. E. Eder, A. Stenzl, A. Hobisch, M. V. Cronauer, G. Bartsch, H. Klocker

**Affiliations:** Department of Urology, University of Innsbruck, Austria.

## Abstract

**Images:**


British Journal of Cancer (1997) 75(12), 1753–1760
© 1997 Cancer Research Campaign

### Expression of transforming growth factors beta-1, beta 2 and beta 3 in human bladder carcinomas

**IE Eder, A Stenzl, A Hobisch, MV Cronauer, G Bartsch and H Klocker**

Department of Urology, University of Innsbruck, A-6020 Innsbruck, Austria

**Summary** We previously detected elevated transforming growth factor beta-1 (TGF-β1) serum levels in patients with invasive bladder carcinomas. In this study, we therefore investigated whether elevated serum levels correlate with enhanced TGF-β expression in human bladder tumours. mRNA levels of TGF-β1, -β2 and -β3 were reduced in bladder tumour tissue to 86%, 68% and 56%, respectively, of the levels in normal urothelium. On the other hand, TGF-β1 protein levels were found to be higher in superficial tumours ($T_a$–$T_1$) (mean level of 0.153 ng $mg^{-1}$) and in invasive $T_2$/$T_3$ tumours (mean level of 0.104 ng $mg^{-1}$) compared with normal urothelium (mean level of 0.065 ng $mg^{-1}$). Invasive $T_4$ tumours, however, contained only low amounts of TGF-β1 (mean level of 0.02 ng $mg^{-1}$). Neither in mean nor in individual patients were serum and tissue TGF-β levels correlated with each other. Cell culture experiments on primary bladder cells revealed a 57% decrease in TGF-β1 mRNA levels in tumour compared with normal epithelial cells. Tumour epithelial cells contained about two times higher levels of TGF-β2 and TGF-β3 mRNA than normal epithelial cells. Fibroblasts expressed about the same amount of TGF-β1 or TGF-β2 as epithelial cells. Yet, fibroblasts released only 19% and 13% of the amount secreted by tumour epithelial cells into the supernatant. TGF-β3, on the other hand, was expressed by fibroblasts with higher levels than by epithelial cells. TGF-β1 was the predominent isoform in bladder tissue and cells at protein as well as on mRNA levels indicating that TGFs-β2 and -β3 are of minor importance in bladder cancer. In summary, there is a lack of correlation between TGF-β serum levels and TGF-β expression in tumour tissue in bladder cancer.

**Keywords:** bladder carcinoma; transforming growth factor beta; tumour tissue; primary bladder cell culture

Transforming growth factors-β (TGF-β) are a family of multifunctional homodimeric polypeptides. Three different isoforms of TGF-β have been found in mammalian cells, termed TGF-β1, -β2 and -β3. The mature 25-kDa peptides of all isoforms are structurally and functionally similar (Roberts and Sporn, 1990), although their regulation of secretion is distinct. TGF-β1 is secreted from most cells as a 225-kDa latent complex. It is assumed that TGF-β2 and -β3 are also synthesized as latent forms; however, detailed knowledge about the structures of these complexes is lacking (Brown et al, 1990). Activation of latent TGF-β by acidification or, more likely, by certain proteases, like plasmin or cathepsin, play a critical role in the bioavailability of TGF-β (Lyons et al, 1988).

TGF-β was regarded as a negative growth regulator because of its potent anti-proliferative effects on many cell types, such as endothelial and epithelial cells and various cell types of haematopoietic origin in vitro (Roberts and Sporn, 1990). In contrast, overexpression of TGF-β1 has been described in several tumours in vivo (Gorsch et al, 1992; Thompson et al, 1992; Steiner et al, 1994) and has also been associated with tumour progression and metastasis (Weidner et al, 1991; Gajdusek et al, 1993). The advantageous effect of this increased TGF-β synthesis on tumour growth is probably caused by autocrine or paracrine activities resulting in increased cell matrix interactions, inhibited immune surveillance or increased angiogenic activity (Kekow et al, 1990; Yang and Moses, 1990; Kehrl, 1991). It has been suggested that in most cases malignant epithelial cell lines have lost their responsiveness to the inhibiting activity of TGF-β (Chang et al, 1993).

In the course of investigations about the role of TGF-β as a potential tumour-promoting factor, elevated TGF-β1 plasma levels have been detected in patients with invasive prostate cancer (Ivanovic et al, 1995). In a similar study, we previously found significantly elevated TGF-β1 serum levels in patients with invasive bladder carcinomas (Eder et al, 1996). In the present study, we investigated whether those elevated TGF-β serum levels correlate with increased TGF-β expression in bladder tumour tissue and whether isoforms differ in their respective expression. In addition, we cultured primary epithelial and fibroblast urothelial cells in order to study the importance of the stromal part in TGF-β expression and secretion.

*Received 24 July 1961*
*Revised 26 November 1961*
*Accepted 4 December 1961*

*Correspondence to:* H Klocker, Department of Urology, University of Innsbruck, A-6020 Innsbruck, Anichstr. 35, Austria

### MATERIALS AND METHODS

### Acid–ethanol extraction of TGF-β1 from bladder tissue

We investigated tissue specimens from 23 patients with transitional cell carcinoma (TCC) and compared them with a group of 22 samples from normal bladder urothelium. Tumour specimens comprised nine superficial ($T_a$, $C_{is}$, $T_1$) and 14 invasive tumours ($T_2$–$T_4$). Normal bladder urothelium was dissected from histologically proven non-malignant areas during radical cystectomy. Tumour samples were taken either during transurethral resection or during radical cystectomy. All samples were immediately frozen in liquid nitrogen. The extraction procedure as described by Roberts et al (1983) was applied in a slightly modified form. The tissue was minced and incubated in a solution of 93% ethanol and 0.23 M hydrochloric acid with gentle overnight shaking at 4°C.

**Figure 1** Primary bladder cells were cultured as described in Materials and methods. Epithelial cells were grown in a serum-free medium (**A**). Fibroblasts, grown in a medium supplemented with 10% FCS, depicted the typical growth morphology pattern (**B**). Both cell types were differentiated not only by light microscopy but also by immunocytochemistry. Epithelial cells were stained with anti-cytokeratin 8–18+19 antibody (**C**), whereas fibroblasts showed no reaction with the same antibody (**D**)

 © Cancer Research Campaign 1997

**Figure 2** Using RT–PCR, we investigated mRNA levels of TGF-β1, -β2 and -β3 in 16 tissue samples derived from human bladder tumours of different stages ($T_a$–$T_4$) and grades ($G_1$–$G_3$) (numbers 10–25) and compared them with nine tissue samples from normal urothelium (numbers 1–9). mRNA levels are indicated as a ratio of TGF-β to GAPDH. Mean values are indicated as horizontal lines

After centrifugation, the supernatant was precipitated with two volumes of ethanol (95%) and four volumes of diethylether for 48 at -20°C. The resulting precipitate was collected by centrifugation and resuspended in phosphate-buffered saline (PBS).

TGF-β1 values were measured by enzyme-linked immunosorbent assay (ELISA; Genzyme) and expressed in ng TGF-β1 mg$^{-1}$ protein (ng mg$^{-1}$). The protein content of the samples was determined according to the method of Bradford (1976).

### Semi-quantitative polymerase chain reaction (PCR)

Total RNA was isolated from frozen tissues or cells using a guanidinium thiocyanate–acid phenol–chloroform (pH 4.0) extraction method. Samples were precipitated with isopropanol, washed with 70% ethanol, dried, dissolved in protease K buffer (50 mM Tris, 20 mM EDTA, 100 mM Sodium chloride and 1% sodium dodecyl sulphate; pH 7.5) and treated with protease K (200 μg ml$^{-1}$) (Sigma-Aldrich, Vienna, Austria) at 55°C for 15 min. Thereafter, the phenol–chloroform extraction procedure was repeated. Finally, RNA was precipitated by isopropanol, washed twice with 70% ethanol, dried and redissolved in diethylpyrocarbonate (DEPC)-treated water. The amount of RNA was determined by measuring the absorbance at 260 nm.

Reverse transcription was performed for 8 min at 20°C, 8 min at 25°C and 30 min at 42°C (four cycles) on 500 ng of RNA in 40 μl containing finnzyme buffer [20 mM, potassium phosphate pH 7.2, 0.2 mM dithiothreitol (DTT), 0.02% Triton X-100, 5% glycerol], 0.5 mM dNTPs, 200 pmol $N_6$-primers, 0.1% β-mercaptoethanol, 0.1 mg ml$^{-1}$ bovine serum albumin (Pharmacia, Vienna, Austria), 39 units ribonuclease inhibitor (Promega) and 10 units finnzyme AMV reverse transcriptase (Biotrade, Margaritella, Vienna, Austria).

PCR was performed with 2 μl of cDNA (diluted in water in order to guarantee a quantitation of PCR fragments in the exponential phase of the reaction) in a final volume of 50 μl containing buffer [2 mM Tris-HCl, pH 7.4, 0.01 mM EDTA, 0.1 mM DTT, 10 mM potassium chloride, 0.01% Triton X-100, 16 μg ml$^{-1}$ bovine serum albumin (BSA), 5% glycerol], 0.2 m$^{m}$ dNTPs, 0.62 units Dyna-Zyme polymerase (Biotrade) and 0.25 μM of each primer. Primers were synthesized on a 381A DNA Synthesizer (Applied Biosystems, Vienna, Austria): glyceraldehyde-3 phosphate dehydrogenase (GAPDH) was used as an internal control: 5′-CACCACCATGGAGAAGGCTGG-3′ (GAPDH 365) and 5′-GTCTAGCTCAGGGATGACCTTG-3′ (GAPDH 735as*); TGF-β1: 5′-AAGTGGATCC ACGAGCCCAA-3′ (TGF-β1) and 5′-GCTGCACTTGCAGGAGCGCAC-3′ (TGF-β1as*); TGF-β2: 5′-AAATGGATACACGAACCCCAA-3′ (TGF-β2) and 5′-GCTGCATTTGCAAGACTTTAC-3′ (TGF-β2as*); TGF-β3: 5′-AAGTGGGTCCATGAACCTAA-3′ (TGF-β3) and 5′-GCTACATTTACAAGACTTCAC-3′ (TGF-β3as*). Antisense primers were fluorescence labelled at the 5′ end as indicated by an asterisk. Synthesis was performed on a thermocycler 60 (Bio med) with 20 s at 94°C, 15 s at 96°C, 1 min at 55°C and 30 s at 73°C (34 cycles). Afterwards, TGF-β (1 μl) and GAPDH (1 μl) samples were mixed with 2.5 μl of formamide and denatured at 90°C for 2 min. DNA fragments were separated electrophoretically in a 6% polyacrylamide gel using the 370A DNA Sequencer (Applied Biosystems). Fluorescence was measured and quantitated using 672A Software 1.2 (Applied Biosystems). Amounts of TGF-β mRNA were normalized against the corresponding amounts of GAPDH mRNA, which is unaffected by TGF-β1 (Edwards et al, 1985; Norgaard et al, 1996). Results are presented as TGF-β – GAPDH ratio. All measurements were performed at least twice.

### Culture of primary bladder cells

### *Primary culture*

Primary cell cultures were established from fresh tissue specimens taken at transurethral resection or radical cystectomy. The tissue was minced into little pieces. The obtained tissue pieces were

© Cancer Research Campaign 1997 

**Figure 3** After acid–ethanol extraction, TGF-β1 protein levels were determined in tissue specimens from 23 bladder cancer patients and 22 specimens from normal urothelium. The histogram shows TGF-β1 values in ng mg$^{-1}$ protein with mean values indicated as horizontal lines. Tumour specimens were divided into groups of different tumour stages: superficial tumours $T_a$–$T_1$; invasive tumours $T_2$–$T_3$; and invasive $T_4$–tumours

**Figure 4** Correlation of serum and tissue TGF-β1 values in individual patients. *r* describes the status of correlation allowed to adhere to the plastic surface of a 25-cm$^2$ culture flask (Costar) and gently covered with Earl's modified Eagle medium (EMEM) supplemented with 20% fetal calf serum (FCS), 1% of a non-essential amino acid solution (Biological Industries), transferrin (1 μg ml$^{-1}$), insulin (1 μg ml$^{-1}$) and penicillin/streptomycin (120 IU ml$^{-1}$, 120 μg ml$^{-1}$). After 2–7 days, when cells began to grow out of the tissue pieces, cultures were incubated in different culture media in order to split epithelial cells and fibroblasts respectively.

### *Culture of epithelial cells*

For the culture of epithelial cells, we used a serum-free MCDB-153 medium (Sigma) supplemented with 1% of a solution of non-essential amino acids (Biological Industries), epidermal growth factor (EGF; 5 ng ml$^{-1}$); Life Technologies, Gibco), bovine pituitary extracts (30 μg ml$^{-1}$; Life Technologies, Gibco), 0.2% bovine serum albumin (Behring, Vienna, Austria), transferrin (10 μg ml$^{-1}$), insulin (1 μg ml$^{-1}$) and penicillin/streptomycin (120 IU ml$^{-1}$, 120 μg ml$^{-1}$). Figure 1A depicts a culture of primary bladder epithelial cells.

### *Culture of fibroblast cells*

Fibroblasts were cultured in EMEM supplemented with 10% FCS, a 1% solution of non-essential amino acids (Biological Industries) and penicillin/streptomycin (120 IU ml$^{-1}$, 120 μg ml$^{-1}$). Figure 1B shows a culture of primary fibroblast cells.

Epithelial cells and fibroblasts were differentiated by both light microscopy, according to the typical growth morphology, and immunocytochemistry, investigating the expression of cytokeratin 8+18+19 (Monosan). Figure 1C and D outline cytokeratin expression in epithelial and fibroblast cells respectively.

### *Collection of cell pellets and culture supernatants*

Cells were harvested with trypsin/EDTA (0.05%, 0.01%), centrifuged at 8000 r.p.m. for 5 min and shock frozen in liquid nitrogen. Cell culture supernatants were taken from 72 h conditioned media, centrifuged to remove cell debris and stored at –20°C. The amount of TGF-β was corrected by the number of cells. Values are presented in ng 10$^{-5}$ cells. In order to avoid false-positive results, TGF-β content of unconditioned media was subtracted from the values measured in cell-conditioned media. We used standard media as described above. EMEM that was used for cultivation of fibroblasts contained 1.3 ng ml$^{-1}$ TGF-β1 and 0.06 pg ml$^{-1}$ TGF-β2, whereas the serum-free MCDB-153, used for cultivation of epithelial cells, contained only 0.06 ng ml$^{-1}$ TGF-β1 and 0.01 pg ml$^{-1}$ TGF-β2.

### Enzyme-linked immunosorbent assay (ELISA)

TGF-β1 and TGF-β2 ELISAs (Genzyme and R&D Systems respectively) were performed according to the manufacturer's instructions. Since only active forms of TGF-β1 and -β2 can be recognized by the antibodies used in the kits, all samples except those received by acid–ethanol extraction were activated by acidification as described previously (Eder et al, 1996).

### Statistical analysis

Probability values were calculated by Kruskal–Wallis and Mann–Whitney *U*-tests. *P*-values < 0.05 were taken as statistically significant.

© Cancer Research Campaign 1997

**Figure 5** mRNA levels of TGF-β1, -β2 and -β3 were determined in a panel of different human primary bladder cell lines. Epithelial cells derived from normal urothelium (BNF) and tumour epithelial cells (BCE) were grown in a special serum-free medium as described in Materials and methods. Fibroblasts derived from normal urothelium (BNE) and tumour-derived fibroblasts (BCF) were cultured in EMEM supplemented with 10% fetal calf serum. mRNA levels were quantitated by RT–PCR and indicated as TGF-β–GAPDH. Mean values are indicated as horizontal lines

### RESULTS

### Expression of mRNA of TGF-β1, -β2 and -β3 in human bladder tissue specimens

Using semi-quantitative PCR, we compared expression of TGF-β1, -β2 and -β3 mRNA in 16 bladder tumour tissue specimens with those in nine samples from normal urothelium. Results are summarized in Figure 2. In normal urothelium, all three TGF-βs were expressed. TGF-β1 mRNA was also present in all 16 tumour samples. On the contrary, TGF-β2 mRNA was only found in 11 and TGF-β3 – mRNA only in 12 tumour samples. TGF-β1 expression with a median TGF-β1 – GAPDH ratio of 0.218 was reduced in tumour tissue compared with normal urothelium with a median TGF-β1 – GAPDH ratio of 0.254. Similarly, TGF-β2 and TGF-β3 mRNA levels were lower in tumour tissue (TGF-β2 = 0.038; TGF-β3 = 0.038) than in normal urothelium (TGF-β2 = 0.054; TGF-β3 = 0.066). Because of the strong variability within the different tissue samples, statistical analysis did not reveal any significant difference in mRNA expression between normal and tumour tissues. The separation of tumour samples into different tumour stages showed that superficial $T_a$ tumours expressed slightly lower amounts of TGF-β mRNAs than invasive tumours. However, there was no significant difference between superficial and invasive tumours.

### TGF-β1 protein levels in human bladder tissue specimens

Tissue specimens were extracted by acid–ethanol. The amounts of TGF-β1 were measured by ELISA and related to total protein in the extracts. As shown in Figure 3, TGF-β1 protein levels were significantly higher in superficial tumours ($T_a$, $T_1$) with 0.153 ng mg$^{-1}$ ($n$ = 9) than in normal urothelium, which contained only 0.065 ng mg$^{-1}$ ($n$ = 22) ($P$ = 0.01). Similarly, invasive $T_2$–$T_3$ tumours had increased TGF-β1 protein levels of 0.104 ng mg$^{-1}$ ($n$ = 11). However, the difference to normal controls and superficial tumours failed to reach statistical significance. Invasive $T_4$ tumours contained significantly lower TGF-β1 protein levels of 0.02 ng mg$^{-1}$ ($n$ = 3) than superficial ($P$ = 0.03) and invasive $T_2$/$T_3$ tumours ($P$ = 0.01) respectively. TGF-β1 levels of $T_4$ tumours were also reduced compared with normal urothelium, but differences were not statistically significant ($P$ = 0.15).

TGF-β1 protein levels were higher in $G_1$/$G_2$ tumours ($n$ = 8) than in poorly differentiated $G_3$ tumours ($n$ = 15) (0.140 ng mg$^{-1}$ and 0.098 ng mg$^{-1}$ respectively) (data not shown). Differences were not statistically significant.

### Correlation of TGF-β1 levels between serum and tissue specimens in individual patients

We investigated whether there is a correlation between preoperative serum TGF-β1 levels and the amount of TGF-β1 in tumour tissue samples from the same patient. However, we could not find any correlation ($r$ = 0.09) (Figure 4).

### Expression of mRNA of TGF-β1, -β2 and -β3 in different bladder cells

We further investigated expression of the three TGF-β isoforms in various human primary bladder cells derived from normal urothelium as well as from bladder tumours of different stages ($T_1$–$T_4$) (Figure 5). Epithelial cells and fibroblasts were separated by selective culture conditions as described in Materials and methods. TGF-β1 was expressed in all 23 cell strains examined. Normal epithelial cells ($n$ = 5) contained higher TGF-β1 mRNA levels with a median TGF-β1 – GAPDH ratio of 0.510 compared with tumour epithelial cells ($n$ = 7), which had a median TGF-β1 – GAPDH ratio of 0.291. However, the difference was not statistically significant. In general, TGF-β1 mRNA levels were higher than both TGF-β2 and TGF-β3.

© Cancer Research Campaign 1997

**Figure 6** The amount of TGF-β1 and -β2 in supernatants from human primary bladder cells was measured by immunoassays. Epithelial cells derived from tumour tissue (BCE) were subdivided into two groups, namely cells derived from superficial ($T_a$–$T_1$) and from invasive tumours ($T_2$–$T_4$). Moreover, supernatants from normal bladder epithelial cells (BNE), normal fibroblast (BNF) and tumour-derived fibroblasts (BCF) were investigated. Mean values are indicated as horizontal lines

TGF-β2, as well as TGF-β3, mRNA levels were higher in tumour epithelial cells ($n$ = 7) (TGF-β2 – GAPDH ratio = 0.053; TGF-β3 – GAPDH ratio = 0.005) compared with normal epithelial cells ($n$ = 5) (TGF-β2) – GAPDH ratio = 0.031; TGF-β3 – GAPDH ratio = 0.003). However, differences were not statistically significant. TGF-β3 expression was very weak. mRNA of TGF-β3 was found in only 15 out of 23 cell strains.

Tumour-derived fibroblasts ($n$ = 7) expressed higher TGF-β3 mRNA levels (TGF-β3 – GAPDH ratio = 0.021) than epithelial cells. By contrast, TGF-β1 and TGF-β2 mRNA levels were almost equal in epithelial cells and fibroblasts. Tumour-derived fibroblasts contained a median TGF-β1 – GAPDH ratio of 0.569 ($n$ = 7) and a median TGF-β2 – GAPDH ratio of 0.028 ($n$ = 7).

### Amount of TGF-β1 and TGF-β2 in cell-conditioned media

Using immunoassays, we evaluated TGF-β1 and TGF-β2 levels in 72 h-conditioned media of several primary bladder cells. Both TGF-β1 and TGF-β2 could only be measured in acid-activated cell culture supernatants (data not shown). This suggests that TGF-β was mostly present in its latent form, as only active TGF-β can be detected by the immunoassays used in this study. Results are presented in Figure 6.

Epithelial cells (BCE) derived from invasive tumours ($T_2$ – $T_4$, $n$ = 4) secreted slightly higher TGF-β1 levels of 4.23 ng $10^{-5}$ cells than epithelial cells derived from superficial tumours ($T_a$–$T_1$, $n$ = 7) (3.79 ng TGF-β1 $10^{-5}$ cells). However, there was no significant difference from normal epithelial cells (BNE), whose conditioned media contained 3.74 ng TGF-β1 $10^{-5}$ cells ($n$ = 7).

Compared with epithelial cells, 0.75 ngTGF-β1 $10^{-5}$ cells and 0.74 ngTGF-β1 $10^{-5}$ cells were detected in supernatants of normal (BNF, $n$ = 7) and tumour-derived fibroblasts (BCF, $n$ = 10) respectively.

Epithelial cells derived from superficial ($n$ = 3) and invasive ($n$ = 2) tumours secreted higher amounts of TGF-β2 (0.8 ng $10^{-5}$ cells) than normal epithelial cells ($n$ = 2) in whose supernatants no TGF-β2 was present. Only very low amounts of 0.08 ng TGF-β2 $10^{-5}$ cells ($n$ = 4) were found in supernatants from tumour-derived fibroblasts. Amounts of TGF-β1 in cell culture supernatants were generally higher than those of TGF-β2.

### DISCUSSION

Overexpression of TGF-β1 has been commonly associated with prostate (Thompson et al, 1992), breast and renal cancer (Gomella et al, 1989, Coombes et al, 1990). In a previous study, we therefore determined TGF-β1 in sera from bladder cancer patients. TGF-β1 was found to be significantly elevated in sera from patients with invasive bladder cancer ($T_2$–$T_4$) compared with serum levels from patients with superficial tumours ($T_a$–$T_1$) and normal control subjects (Eder et al, 1996).

In this study, we were interested in whether elevated TGF-β serum levels correlate with increased TGF-β expression in bladder tumours. However, we found reduced expression of mRNA of all three TGF-β isoforms, TGF-β1, -β2 and -β3, in bladder tumour samples compared with normal urothelium. There was neither a correlation of TGF-β mRNA expression with tumour stages ($T_a$–$T_4$) nor with tumour grades ($G_1$–$G_3$). These results were somehow unexpected, considering the increased serum levels in bladder cancer patients. On the other hand, reduced TGF-β1 mRNA has already been demonstrated in high-grade human bladder tumours (Coombs et al, 1993; Miyamoto et al, 1995).

Investigating TGF-β1 protein levels in bladder tissue, we found TGF-β1 elevated significantly in superficial forms of bladder cancer ($T_a$–$T_1$) and moderately in invasive bladder carcinomas of stages $T_2$ and $T_3$ compared with normal urothelium. By contrast, TGF-β1 protein levels were significantly decreased in invasive $T_4$

© Cancer Research Campaign 1997

tumours. Similarly, TGF-β1 levels were lower in poorly differentiated in comparison with well and moderately differentiated tumours. It is striking that TGF-β1 protein levels in tumour tissue are in inverse proportion to serum levels. High TGF-β1 serum levels in patients with invasive bladder carcinomas correlate with a small increase or a significant decrease of protein levels in $T_2$–$T_3$ and $T_4$ tumour tissue, respectively, whereas low TGF-β1 serum levels in patients with superficial bladder tumours correlate with a significantly elevated protein level in tumour tissue. One can assume that accumulation of TGF-β in superficial tumours slows down tumour progression through its growth-suppressive effects as supposed by Miyamoto et al (1995). An explanation for the small increase or decrease, respectively, in invasive tumours may be that $T_4$ tumours and, to a lesser extent, also $T_2$ and $T_3$ tumours immediately release TGF-β into the surrounding tissue and the circulation in order to exhibit immune suppression and stimulate metastatic spread, whereas in the superficial tumours, TGF-β is accumulated in the tumour. Efficient access to the circulation is enabled by the extended vascularization of invasive tumours.

Comparison of TGF-β serum and tumour levels of individual patients also revealed no direct correlation with each other, indicating that the expression level in the tissue is not crucial for the final serum concentration. Other parameters, such as release rate, blood supply of the tumour and tumour mass, are obviously more important.

In the tissue samples we investigated, there was also no correlation between mRNA and protein levels. This finding is in accordance with previous studies, which already indicated that there is no evidence for a direct correlation between TGF-β1 protein and mRNA levels. Similar discrepancies have already been described in the mouse embryo (Pelton et al, 1991). Differential expression seems to be caused by post-transcriptional regulation of expression through a 5′ untranslated sequence in the TGF-β mRNA (Kehrl et al, 1986; Kim et al, 1992). On the basis of our results, we conclude that neither mRNA nor protein levels found in tumour tissue provide a conclusive explanation for increased TGF-β1 levels in the serum of patients with invasive bladder cancer.

An important question is whether the stromal part of the tumour plays an important role in TGF-β production. It is well established that the TGF-βs are secreted proteins; thus, certain cells may synthesize and release TGF-β into the extracellular matrix of adjacent cells that have the potential to respond. Both autocrine and paracrine factors, which are produced by epithelial and stromal cells, may play an important role in the local growth control, since it is known that epithelium loses its growth capacity when separated from the stroma (Kooistra et al, 1995). Some malignant tumours may use TGF-β released from their environment for enhancing their invasiveness and metastatic behaviour, while at the same time being resistant to the growth-suppressive effect of TGF-β. Exposure of tumour cells to exogenous TGF-β has been shown to stimulate tumour cell invasion in vivo and metastatic potential in vitro (Welch et al, 1990). In order to investigate the contribution of different cell types to protein levels in the tissue, we cultured human primary bladder cells separating epithelial from fibroblast cells by different culture conditions.

Our measurements in samples of cultured primary cells revealed remarkable differences among the TGF-β isoforms concerning their expression and secretion. TGF-β1 mRNA was expressed in lower levels in tumour-derived compared with normal epithelial cells, whereas TGF-β1 protein secretion into the culture medium was almost equal between these two cell types. On the other hand, TGF-β2 and TGF-β3 mRNAs were found to be slightly higher expressed in tumour epithelial cells than in normal epithelial cells. Accordingly, higher amounts of TGF-β2 were found in cell culture supernatants of tumour-derived compared with normal epithelial cells. Considering that the absolute amounts of secreted TGF-β2 are about one-tenth of the amount of secreted TGF-β1, the differences in TGF-β2 release are probably of minor importance.

In cultured fibroblasts, about the same amount of TGF-β mRNAs as in epithelial cells as measured. This points out the important role of the interaction between epithelium and stroma, and lets us suppose that fibroblasts would also secrete considerable amounts of TGF-β. Yet, unexpectedly, fibroblasts released only small amounts of TGF-β1 and TGF-β2 into the supernatant. This may be the result of a lower efficiency of translation of TGF-β mRNA in fibroblasts, but it is also possible that a great part of the produced TGF-β is immediately exhausted by the cells in culture as a result of autocrine stimulation.

In summary TGF-β is assumed to be overexpressed in superficial bladder cancer compared with invasive forms of bladder cancer, indicating that expression is inversely correlated with TGF-β serum levels. Additionally, cell culture experiments revealed that stromal cells play a role in TGF-β production. Cocultures of epithelial and fibroblast cells should bring further information about the interaction between epithelium and stroma.

### ACKNOWLEDGEMENTS

We thank R. Höllinger and G Sierek for technical assistance. This work is supported in part by a grant of the Jubiläumsfonds der Österreichischen Nationalbank, project number 5499.

### REFERENCES

Bradford MM (1976) A rapid and sensitive method for the quantitation of microgram quantities of protein utilizing the principle of protein-dye binding. *Anal Biochem* **72**: 248–254

Brown PD, Wakefield LM, Levinson AD and Sporn MB (1990) Physicochemical activation of recombinant latent transforming growth factor-betas 1, 2 and 3. *Growth Factors* **3**: 35–43

Chang H-L, Gillett N, Figari I, Lopez AR, Palladino MA and Derynck R (1993) Increased transforming growth factor β expression inhibits cell proliferation *in vitro*, yet increases tumorigenicity and tumour growth of Meth A sarcoma cells. *Cancer Res* **53**: 4391–4398

Coombes RC, Barrett-Lee P and Luqmani Y (1990) Growth factor expression in breast tissue. *J Steroid Biochem Mol Biol* **37**: 833–836

Coombs LM, Pigott DA, Eydmann ME, Proctor AJ and Knowles MA (1993) Reduced expression of TGFβ is associated with advanced disease in transitional cell carcinoma. *Br J Cancer* **67**: 578–584

Eder IE, Stenzl A, Hobisch A, Cronauer MV, Bartsch G and Klocker H (1996) Transforming growth factor-β types 1 and 2 in serum and urine from patients with bladder carcinoma. *J Urol* (in press)

Edwards DR, Parfett CL and Denhardt DT (1985) Transcriptional regulation of two serum-induced RNAs in mouse fibroblasts: equivalence of one species to B2 repetitive elements. *Mol Cell Biol* **5**: 3280–3288

Gajdusek CM, Luo Z and Mayberg MR (1993) Basic fibroblast growth factor and transforming growth factor beta-1: synergistic mediators of angiogenesis in vitro. *J Cell Physiol* **157**: 133–144

Gomella LG, Sargent ER, Wade TP, Anglard P, Linehan WM and Kasid A (1989) Expression of transforming growth factor alpha in normal human adult kidney and enhanced expression of transforming growth factors alpha and beta 1 in renal cell carcinoma. *Cancer Res* **49**: 6972–6975

Gorsch SM, Memoli VA, Stukel TA, Gold LI and Arrick BA (1992) Immunohistochemical staining for TGFβ1 associates with disease progression in human breast cancer. *Cancer Res* **52**: 6949–6952

Ivanovic V, Melman A, Davis-Joseph B, Valvic M and Geliebter J (1995) Elevated plasma levels of TGF-β1 in patients with invasive prostate cancer. *Nature Med* **1**: 282–284

© Cancer Research Campaign 1997

Kehrl JH (1991) Transforming growth factor-β: an important mediator of immunoregulation. *Int J Cell Cloning* **9**: 439–450

Kehrl JH, Wakefield LM, Roberts AB, Jakowlew S, Alvarez-Mon M, Derynck R, Sporn MB and Fauci AS (1986) Production of transforming growth factor beta by human T lymphocytes and its potential role in the regulation of T cell growth. *J Exp Med* **163**: 1037–1050

Kekow J, Wachsman W, McCutchan JA, Cronin M, Carson DA and Lotz M (1990) Transforming growth factor β and noncytopathic mechanisms of immunodeficiency in human immunodeficiency virus infection. *Proc Natl Acad Sci USA* **87**: 8321–8325

Kim S-J, Park K, Koeller D, Kim KY, Wakefield LM, Sporn MB and Roberts AB (1992) Post-transcriptional regulation of the human transforming growth factor-β1 gene. *J Biol Chem* **267**: 13702–13707

Kooistra A, van den Eijnden Raaij AJM, Klaij IA, Romijn JC and Schröder FH (1995) Stromal inhibition of prostatic epithelial cell proliferation not mediated by transforming growth factor beta. *Br J Cancer* **72**: 427–434

Lyons RM, Keski-Oja J and Moses HL (1988) Proteolytic activation of latent transforming growth factor-beta from fibroblast-conditioned medium. *J Cell Biol* **106**: 1659–1665

Miyamoto H, Kubota Y, Shuin T, Torigoe S, Dobashi Y and Hosaka M (1995) Expression of transforming growth factor-beta 1 in human bladder cancer. *Cancer* **75**: 2565–2570

Norgaard P, Spang-Thomsen M and Poulsen HS (1996) Expression and autoregulation of transforming growth factor β receptor mRNA in small-cell lung cancer cell lines. *Br J Cancer* **73**: 1037–1043

Pelton RW, Saxena B, Jones M, Moses HL and Gold LI (1991) Immunohistochemical localization of TGFβ1, TGFβ2 and TGFβ3 in the mouse embryo: expression patterns suggest multiple roles during embryonic development. *J Cell Biol* **115**: 1091–1105

Roberts AB and Sporn MB (1990) The transforming growth factor-βs. In *Peptide Growth Factors and Their Receptors I*, 95th edn, Roberts AB and Sporn MB (eds), pp. 419–472. Springer Verlag: Heidelberg, Germany.

Roberts AB, Anzano MA, Meyers CA, Wideman J, Blacher R, Pan Y-CE, Stein S, Lehrman SR, Smith JM, Lamb LC and Sporn MB (1983) Purification and properties of a type β transforming growth factor from bovine kidney. *Biochemistry* **22**: 5692–5698

Steiner MS, Zhou Z-Z, Tonb DC and Barrack ER (1994) Expression of transforming growth factor-β1 in prostate cancer. *Endocrinology* **135**: 2240–2247

Thompson TC, Truong LD, Timme TL, Kadmon D, McCune BK, Flanders KC, Scardino PT and Hee Park S (1992) Transforming growth factor β1 as a biomarker for prostate cancer. *J Cell Biochem* **16H**: 54–61

Weidner N, Semple JP, Welch WR and Folkman J (1991) Tumor angiogenesis and metastasis – correlation in invasive breast carcinoma. *N Engl J Med* **324**: 1–8

Welch DR, Fabra A and Nakajima M (1990) Transforming growth factor β stimulates mammary adenocarcinoma cell invasion and metastatic potential. *Proc Natl Acad Sci USA* **87**: 7678–7682

Yang EY and Moses HL (1990) Transforming growth factor β1-induced changes in cell migration, proliferation and angiogenesis in the chicken chorioallantoic membrane. *J Cell Biol* **111**: 731–741

© Cancer Research Campaign 1997